# Machine Learning Creates a Simple Endoscopic Classification System that Improves Dysplasia Detection in Barrett's Oesophagus amongst Non-expert Endoscopists

**DOI:** 10.1155/2018/1872437

**Published:** 2018-08-29

**Authors:** Vinay Sehgal, Avi Rosenfeld, David G. Graham, Gideon Lipman, Raf Bisschops, Krish Ragunath, Manuel Rodriguez-Justo, Marco Novelli, Matthew R. Banks, Rehan J. Haidry, Laurence B. Lovat

**Affiliations:** ^1^Department of Gastroenterology, University College London Hospitals NHS Foundation Trust, London, UK; ^2^Research Department for Tissue & Energy, Division of Surgery & Interventional Science, University College London, London, UK; ^3^Department of Industrial Engineering, Jerusalem College of Technology, Jerusalem, Israel; ^4^Department of Gastroenterology, University Hospitals Leuven, Leuven, Belgium; ^5^Department of Gastroenterology, Queen's Medical Centre, Nottingham, UK; ^6^Department of Histopathology, University College London Hospitals NHS Foundation Trust, London, UK

## Abstract

**Introduction:**

Barrett's oesophagus (BE) is a precursor to oesophageal adenocarcinoma (OAC). Endoscopic surveillance is performed to detect dysplasia arising in BE as it is likely to be amenable to curative treatment. At present, there are no guidelines on who should perform surveillance endoscopy in BE. Machine learning (ML) is a branch of artificial intelligence (AI) that generates simple rules, known as decision trees (DTs). We hypothesised that a DT generated from recognised expert endoscopists could be used to improve dysplasia detection in non-expert endoscopists. To our knowledge, ML has never been applied in this manner.

**Methods:**

Video recordings were collected from patients with non-dysplastic (ND-BE) and dysplastic Barrett's oesophagus (D-BE) undergoing high-definition endoscopy with *i-Scan* enhancement (PENTAX®). A strict protocol was used to record areas of interest after which a corresponding biopsy was taken to confirm the histological diagnosis. In a blinded manner, videos were shown to 3 experts who were asked to interpret them based on their mucosal and microvasculature patterns and presence of nodularity and ulceration as well as overall suspected diagnosis. Data generated were entered into the WEKA package to construct a DT for dysplasia prediction. Non-expert endoscopists (gastroenterology specialist registrars in training with variable experience and undergraduate medical students with no experience) were asked to score these same videos both before and after web-based training using the DT constructed from the expert opinion. Accuracy, sensitivity, and specificity values were calculated before and after training where *p* < 0.05 was statistically significant.

**Results:**

Videos from 40 patients were collected including 12 both before and after acetic acid (ACA) application. Experts' average accuracy for dysplasia prediction was 88%. When experts' answers were entered into a DT, the resultant decision model had a 92% accuracy with a mean sensitivity and specificity of 97% and 88%, respectively. Addition of ACA did not improve dysplasia detection. Untrained medical students tended to have a high sensitivity but poor specificity as they “overcalled” normal areas. Gastroenterology trainees did the opposite with overall low sensitivity but high specificity. Detection improved significantly and accuracy rose in both groups after formal web-based training although it did it reach the accuracy generated by experts. For trainees, sensitivity rose significantly from 71% to 83% with minimal loss of specificity. Specificity rose sharply in students from 31% to 49% with no loss of sensitivity.

**Conclusion:**

ML is able to define rules learnt from expert opinion. These generate a simple algorithm to accurately predict dysplasia. Once taught to non-experts, the algorithm significantly improves their rate of dysplasia detection. This opens the door to standardised training and assessment of competence for those who perform endoscopy in BE. It may shorten the learning curve and might also be used to compare competence of trainees with recognised experts as part of their accreditation process.

## 1. Introduction

Invasive oesophageal adenocarcinoma (OAC) is associated with a poor overall 5-year survival of 15% [1]. Barrett's oesophagus (BE) is the only identifiable premalignant lesion. Endoscopic surveillance of BE is performed to detect OAC at a pre-invasive stage that is likely to be amenable to curative treatment. Current surveillance strategies typically rely on white light endoscopy (WLE) to obtain random four-quadrant biopsies every 2 cm through the BE segment [2]. This approach samples less than 5% of the Barrett's epithelium and is therefore prone to missing early lesions [3].

A novel endoscopic image enhancement technology, *i-Scan* (PENTAX HOYA, Japan), has been developed to improve lesion recognition in the gastrointestinal tract. *i-Scan* utilises post processing light filtering technology to enhance different elements on the mucosa and microvasculature. We recently published the first formal classification system for *i-Scan* in the upper gastrointestinal tract [4]. There is also growing evidence to suggest increased accuracy of dysplasia detection using *i-Scan* in the assessment of colorectal polyps [5–7].

Machine learning (ML) is a branch of data mining that applies mathematical models to generate computerised algorithms. These can create novel prediction models. ML involves a computer “learning” important features of a dataset to enable predictions about other, unseen, data. A potential application would be to separate subjects into two (but sometimes more) categories based on the attributes measured. This could then be used to create predictive models about which subjects have a disease.

Over the past 10 years, ML has become increasingly used within the field of medicine. For instance, ML has already been applied to improve detection of ovarian and breast cancers using ultrasonography [8, 9]. Models use patient-specific information to predict a medical outcome or to help support doctors in making a diagnosis [10]. ML also has potential training applications. An example includes IBM's WatsonPaths project (IBM Research), which has been developed to improve training and diagnostic skills amongst medical students at the Cleveland Clinic (http://www.research.ibm.com). Other examples include ML to prognosticate in melanoma, predict susceptibility for cerebrovascular disease, risk of recurrence of breast cancer, and diagnosis of thyroid disease [11–13]. Interestingly, these systems often outperform the diagnostic abilities of specialists in each field.

Currently, there is no standardised method of assessing the competence of endoscopists who perform endoscopy in patients with BE. It is perhaps therefore not surprising that there is considerable variability in diagnostic competence amongst endoscopy trainees across Europe with almost one-third admitting to feeling uncertain when performing endoscopy in BE [14]. A training tool to improve endoscopic lesion recognition in BE is therefore desirable.

The aims of this study were:

- To evaluate the accuracy of expert endoscopists to detect dysplasia in patients with BE using *i-Scan*.
- Using the data generated by expert endoscopists to generate a simple automatic machine-based algorithm to help detect dysplasia in patients with BE.
- To assess if training non-expert endoscopists using this algorithm could improve their detection of dysplasia.

## 2. Methods

### 2.1. i-Scan Technology

**Figure 1:**
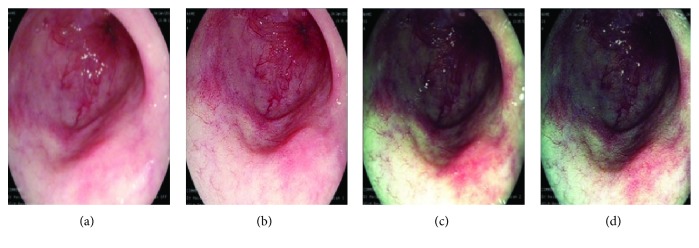
Endoscopic appearances of a flat segment of nondysplastic Barrett's in each separate i-Scan mode. (a) - WLE, (a) i-Scan 1, (c) i-Scan 2, and (d) i-Scan 3.

i-Scan is a novel endoscopic post processing light filtering technology (PENTAX HOYA Corporation). Software algorithms with real-time image mapping technology are embedded with an EPKi processor. The EPKi processor enables resolution above high-definition television (HDTV) standard with a resolution of approximately 1.25 megapixels per image. Using distinct digital filters, i-Scan offers post processing of images to allow the endoscopist to make additional analyses. There are three image enhancement modes, which are controlled by pressing a button on the endoscope handpiece (Figure 1):

i. Surface enhancement (i-Scan 1): enhancement of the structure through recognition of the edges.
ii. Contrast enhancement (i-Scan 2): enhancement of depressed areas and differences in structure through the presentation of low-density areas.
iii. Surface and tone enhancement (i-Scan 3): enhancement tailored to individual organs through modification of the combination of red, green and blue (RGB) light components for each pixel.

### 2.2. Classification System

Classification systems using narrow band imaging (NBI, Olympus®) in patients with BE have already been published [15–19]. Broadly speaking, these take into account mucosal and microvascular patterns to predict dysplasia in BE [20]. A recent multi-national consortium of NBI experts (BING consortium) developed a consensus-driven NBI classification system based on 60 NBI magnification images with encouraging results [21]. In this study, the mucosa was classified as normal (circular, ridged, or tubular pattern) or abnormal (absent or irregular patterns) and the vascular pattern as normal (regular vessels with normal or long branching patterns) or abnormal (focally or diffusely distributed vessels following abnormal mucosal architecture). Dysplasia detection was achieved with an accuracy of 85%, (sensitivity 80%, specificity 88%) with substantial interobserver agreement (*κ* = 0.681).

Acetic acid (ACA) chromoendoscopy is increasingly being used to enhance the endoscopic detection of Barrett's dysplasia [22]. When sprayed onto Barrett's mucosa, an acetowhitening reaction occurs such that areas of dysplasia lose their acetowhitening effect faster than areas without dysplasia. This phenomenon helps facilitate targeted biopsies of the Barrett' mucosa. We previously used data from the BING consortium to validate a classification system using i-Scan magnification endoscopy and ACA chromoendoscopy reporting similar outcomes (accuracy 83%) with substantial interobserver agreement (*κ* = 0.69) [4]. We graded lesions based on mucosal (M) and vascular (V) patterns as follows:

i. M1: regular circular or villous pits.
ii. M2: distorted or irregular pits or featureless mucosa.
iii. V1: regular and uniform vessels.
iv. V2: irregular, dilated tortuous vessels.

**Figure 2:**
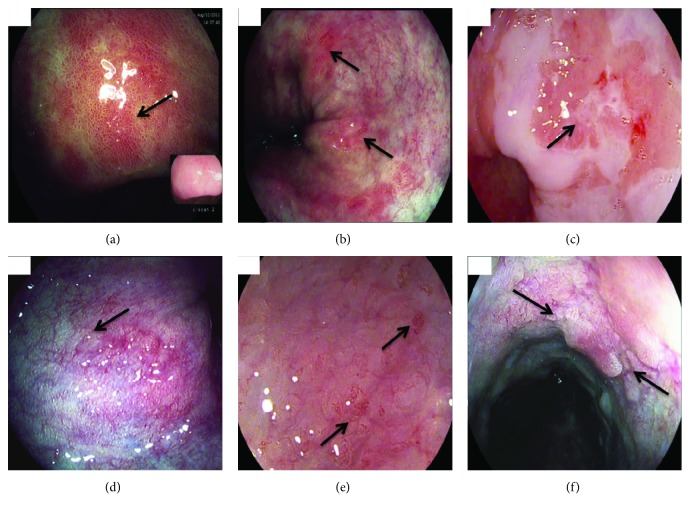
Endoscopic appearances of different mucosal, microvascular patterns, and architecture of Barrett's segment using i-Scan. Arrows denote particular areas of interest. (a) M1: regular circular, oval pits; (b) M1: villous/gyrus ridged pits; (c) M2: distorted, featureless mucosa with surface ulceration; (d) V1: regular uniform vessels; (e) V2: irregular, “corkscrew-like” vessels; (f) nodularity with surrounding distortion of the normal mucosal surface pattern.

Expert endoscopists used the same classifications in the current study. We also took into consideration the presence of nodularity and ulceration (Figure 2). We believed this to be important as both of these features are associated with higher rates of dysplasia in patients with BE and can be easily recognised at endoscopy [23].

### 2.3. Data Mining and Machine Learning

Data mining is the process of selecting, exploring, and modelling large amounts of data in order to discover unknown patterns or relationships [24].

Several types of data classification algorithms exist to classify data into two or more categories. We used decision trees as they output rules that are easily understood by doctors. We entered the endoscopic assessments of three expert endoscopists (Matthew R. Banks, Raf Bisschops, and Rehan J. Haidry) into the “WEKA” ML package to help construct the predictive model [25].

### 2.4. Patients

This study was a UK Health Regulatory Agency (HRA) approved national clinical trial (REC reference 08/H0808/8, study number 08/0018). Patients undergoing surveillance endoscopy for BE were invited to participate. All provided written informed consent. They were asked to stop any anticoagulant therapy for 7 days before endoscopy. Patients in whom acquisition of oesophageal biopsies was contraindicated (coagulopathy, inability to stop anticoagulants, or oesophageal varices) were excluded.

### 2.5. Endoscopy and Recording

High-definition (HD) video recordings were collected from 40 patients at University College London Hospital over an 18-month period. All patients were taking proton pump inhibitors.

Endoscopy was performed using a PENTAX EG-2990i or EG29-i10 HD video gastroscope (PENTAX HOYA, Japan). All procedures were performed by one of three endoscopists with a specialist interest in BE (Laurence B. Lovat, Matthew R. Banks, Rehan J. Haidry). Patients were given conscious sedation using intravenous midazolam and fentanyl. In cases of excessive oesophageal peristalsis, an antispasmodic agent such as hyoscine*-N-*butylbromide (Buscopan) was administered intravenously.

Once the endoscopist had selected an area of interest, the area was cleaned with sterile water. A mucolytic agent such as “N-acetylcysteiene” was used to improve mucosal clarity. The endoscopist switched between white light endoscopy and each separate “i-Scan” mode ensuring each was recorded for 10 seconds. A corresponding biopsy was then taken from all recorded areas to confirm the histological diagnosis. In 12 cases, 3% (ACA) was also applied to the BE segment prior to biopsy. In these cases, the same protocol for recording was used both before and after the application of ACA. ACA was applied at the discretion of the endoscopist. The endoscopist did not classify recordings in real time. Once complete, patients continued to routine endoscopy including acquisition of further biopsies for clinical purposes if relevant.

### 2.6. Histology

All biopsy specimens were reviewed by one of two expert gastrointestinal pathologists (Manuel Rodriguez-Justo and Marco Novelli) who were blinded to endoscopists' interpretations. All biopsies were reported according to the revised Vienna classification [26]. Cases of suspected dysplasia were reviewed by both pathologists in line with current recommendations [27].

### 2.7. Postendoscopy Assessment

Recordings were downloaded to a secure memory drive after the procedure. Recordings were edited using iMovie (Apple Inc.®) to construct individual video files lasting up to 60 seconds that included recordings of each separate “i-Scan” mode. Once the corresponding histology had been reviewed, information was anonymised and entered into a structured database. Later, all videos were transferred to an encrypted memory stick for each expert to review.

### 2.8. Expert Review and Interpretation of Videos

All videos were reviewed blindly by three expert endoscopists (Matthew R. Banks, Raf Bisschops, and Rehan J. Haidry). All were part of the group that developed our i-Scan classification system for dysplasia detection in BE [4]. They were required to provide the following information for each video: M and V scores, presence of nodularity, and ulceration and prediction of histological diagnosis (ND-BE or D-BE). They were also asked to score how certain they were of the diagnosis on a scale of 1–6 (1 completely uncertain, 6 completely certain).

### 2.9. Construction of a Decision Tree for Dysplasia Prediction

Data generated was entered into the WEKA package. This was then used to construct the simplest DT for dysplasia prediction that gave a high accuracy but which was also easy enough to teach to non-expert endoscopists.

### 2.10. Non-expert Review and Interpretation of Videos

Two groups of non-expert endoscopists took part in the study. The first group comprised specialist registrars (SpRs) in gastroenterology who had varying levels of experience in endoscopy and BE assessment. The second group was final year medical students who had no prior experience in either endoscopy or BE.

Both groups were invited to visit a website that hosted all the videos, which were presented in a random fashion. When scoring videos before training, both groups were only asked to record their overall suspected diagnosis and how certain they were of this. Each individual scorer had their own unique access to the website and was unaware of the scoring given by other participants. Once all videos had been scored, individuals were given online access to a 4-minute training presentation. This presentation used a different set of endoscopic videos to highlight the different mucosal and vascular patterns seen both with and without ACA. At the end of this video, the decision tree for dysplasia prediction constructed from expert opinion was presented. The training presentation could be repeated as many times as individuals wished. After training, both groups then scored the original 40 videos. In addition to the suspected diagnosis and certainty, both groups were also asked to record their interpretation of the M and V scores as well as the presence of nodularity or ulceration.

### 2.11. Statistical Analysis

Accuracy, sensitivity, and specificity values were calculated before and after training. Student's paired *t*-test was used to assess differences, and *p* < 0.05 was considered to be statistically significant.

## 3. Results

Video recordings from 40 patients were collected. Twelve had an endoscopy performed using ACA with recordings collected both before and after. Biopsy samples obtained from 23 cases revealed ND-BE and 17 showed D-BE (low-grade dysplasia, 4; high-grade dysplasia, 12; intramucosal cancer, 1). In the cases in which ACA was used, 5 had D-BE (low-grade dysplasia, 1; high-grade dysplasia, 3; intramucosal cancer, 1) and 7 had ND-BE.

### 3.1. Dysplasia Prediction amongst Experts

The mean sensitivity and specificity of dysplasia prediction amongst experts were 88% and 86%, respectively (Table 1). The mean sensitivity of detecting D-BE increased to 93% in the 12 patients who also underwent ACA chromoendoscopy, but this difference was not statistically significant (*p* = 0.5). Negative predictive values (NPV) were calculated for a hypothetical endoscopic surveillance population in which dysplasia is present in 5% of attendees.

### 3.2. Construction of a Decision Tree for Dysplasia Prediction

**Figure 3:**
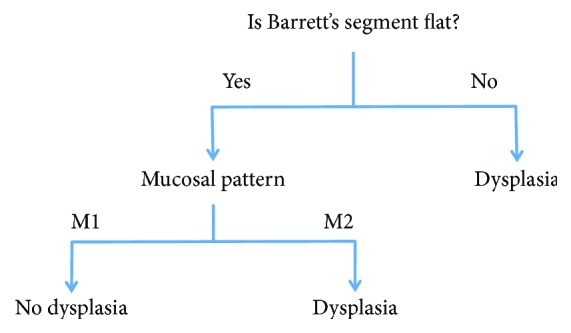
Decision tree constructed from expert opinion that was used to train non-expert endoscopists. M1: regular circular (oval) or villous/gyrus ridged pits; M2: distorted irregular pits or featureless mucosa. Flat means absence of endoscopically visible nodularity or ulceration.

Addition of ACA chromoendoscopy did not lead to a significant difference in dysplasia prediction. The decision tree was therefore constructed without considering experts' answers after ACA was used. As each expert interpreted videos differently, we hypothesised that a model that aggregated their opinions would be helpful. The decision tree was based on aggregated data from all 3 experts equally. Thus, all the information for all dysplastic lesions was aggregated and the machine identified the simplest way to classify based on combining all possible information. The same process was used for all nondysplastic lesions. This is a validated approach in data mining [28]. The final DT is shown in Figure 3.

According to this tree, the most important attribute (i.e. the root of the tree) was the presence or absence of nodularity or ulceration. If there is nodularity or ulceration, according to our proposed DT, the lesion in question is dysplastic. If, however, the Barrett's segment is flat, only then should the assessment of the mucosal pattern be made. When normal (M1), there is no dysplasia, but when abnormal (M2), the lesion in question should be considered dysplastic. This DT, which combined all the experts' assessments led to an overall accuracy for dysplasia prediction of 92%, sensitivity 97%, and specificity of 88%. This was higher than the accuracy of any individual expert.

### 3.3. Dysplasia Prediction amongst Non-experts

In total, 13 SpRs and 9 medical students analysed all 40 videos both before and after training (Table 2). Mean and standard deviation for the accuracy of dysplasia prediction before training in each group was 65% ± 9.5% and 53% ± 7.5%, respectively. After training, the mean accuracy amongst SpRs increased to 68% ± 9.0% (*p* = 0.07) and for medical students to 63% ± 8.9% (*p* = 0.0005). When analysed together, the average accuracy amongst both groups before and after training increased significantly from 60% to 66% (*p* = 0.0005).

The sensitivity of dysplasia prediction amongst SpRs significantly increased after training from 71% ± 13.1% to 83% ± 12.0% (*p* < 0.0001). In contrast, the sensitivity of dysplasia prediction amongst medical students did not increase significantly after training (83% before training, 84% after training; *p* = 0.44).

There was no change in specificity of dysplasia prediction amongst SpRs after training (60% before training versus 57% after, *p* = 0.2). In contrast, the specificity of dysplasia prediction improved dramatically after medical student training from 31% to 49% (*p* < 0.0001).

## 4. Discussion

By using data from three expert endoscopists, a simple classification system derived from machine learning technologies predicted D-BE correctly in 96% of cases with a mean sensitivity and specificity of 93% and 99%. This is better than any single expert's analysis. Although data mining techniques have been used to predict medical outcomes [29] and have also been proposed to help train doctors [30], to our knowledge, machine learning has never been actually applied to medical student teaching and certainly not to improve the endoscopic detection of pre-malignant lesions in the gastrointestinal tract. The only reference in world literature in using machine learning in this way that we could find was to train helicopter pilots [31].

The accuracy of the classification system generated from this small dataset is comparable to select published data using NBI which demonstrated a sensitivity of dysplasia/early cancer detection of 87% both with and without ACA [32].

In contrast to existing classification systems, which are often complex, our proposed algorithm, which was entirely machine generated, is relatively simple [15–19]. The first question is whether the BE segment is flat. Only if nodularity or ulceration is not seen does the system require the user to interpret mucosal architecture. Microvascular patterns can be completely ignored. This is potentially useful, as during the development of our i-Scan classification system of BE dysplasia, we found that experts usually found interpretation of different microvascular patterns more difficult than mucosal surface patterns [4]. The simple machine learning-derived algorithm therefore lends itself well to training.

For non-expert endoscopists, high sensitivity of dysplasia detection is crucial, to ensure adequately targeted biopsies. Before training, medical students, who had no previous experience, had a higher sensitivity of dysplasia detection (83%) than SpRs with varying levels of experience (71%). However, specificity amongst medical students was considerably lower than SpRs (31% versus 60%). This is probably because students were more likely to “overcall” normal areas whereas SpRs did the opposite. Interestingly, after web-based training, specificity rose significantly amongst medical students without loss of sensitivity and significant improvement in overall detection. In contrast, specificity amongst SpRs fell slightly after training (57%) but sensitivity improved significantly (83%) as did the overall accuracy of dysplasia detection albeit the difference reached was not statistically significant. From this, we can conclude that after training, students were more confident in making a diagnosis of ND-BE without loss of sensitivity at detecting D-BE whist for SpRs, the sensitivity at detecting D-BE improved significantly without loss of specificity. However, neither group was able to reach the accuracy of the experts. Reassuringly, when both groups of non-experts were combined, the diagnostic accuracy, sensitivity, and specificity improved significantly after training.

Preservation and incorporation of valuable endoscopic innovations (PIVI) define thresholds that are used to direct endoscopic technology development towards resolving important clinical issues in endoscopy. In response to the limitations of endoscopic surveillance of BE that are currently practiced, the American Society for Gastrointestinal Endoscopy (ASGE) published a PIVI to address real-time imaging of BE [33]. They concluded that to eliminate the need for random mucosal biopsies in BE, any imaging technology with targeted biopsies should have a sensitivity of 90% or greater, a specificity of 80%, and a negative predictive value of at least 98%. Expert assessment did reach the PIVI, but despite improvement in sensitivity and specificity when non-experts used the DT, it did not reach the PIVI standards. It may be useful to expand the current dataset and number of non-experts to evaluate this further.

A similar web-based educational tool to improve the detection and delineation of Barrett's oesophagus-related neoplasia (BORN) has also been published in abstract format [34]. In this study, endoscopy recordings from patients with BORN and non-dysplastic BE were shown to 3 expert endoscopists. After experts had used specialist software to delineate BORN lesions, 68 assessors from the USA and Europe (trainee, junior, and senior gastroenterologists) were asked to detect and delineate these in 4 sets of 20 videos (48 BORN, 32 non-dysplastic) with online training and tailored feedback after each set. Detection and delineation scores significantly increased over the 4 sets (detection score increase: 11%, median delineation score increase: 52%; *p* < 0.0001) with no significant difference between trainees, juniors, and seniors. After removal of 55 videos that were classed as being either “too easy” or “too difficult,” the study was repeated for a new group of 121 assessors across the USA, Canada, and Europe with similar outcomes across all levels of seniority (detection score increase: 26%, median delineation score increase: 77%; *p* < 0.0001). Although the results of this study are not directly comparable with ours, its findings are strengthened by the considerably greater number of videos and assessors with varying levels of experience across multiple centres worldwide that were studied. Until the methods and results of this study are fully published, it remains unclear what exact training was delivered after consecutive video sets and if a classification system was presented to assessors or not.

The novelty of this study from a data mining perspective is that our DT, which was based on expert knowledge, is being used not to improve diagnosis but as an aid to training. To date, DT algorithms have been used as diagnostic support tools as experts can typically easily understand the simple rules generated [10]. In contrast, the approach here is to use DT's to create a logical process that doctors in training can use to assess a new clinical scenario, in this case, unfamiliar endoscopic findings. The reason that DT's are so suited to this purpose is that they are generated to identify the most important features first. Accuracy of diagnosis could be improved in our dataset by using more complex trees. This would defeat the purpose of creating a simple tool which can be used to rapidly train junior doctors. Clearly, this brief training does not elevate the trainees to the level of expert, but it does significantly enhance competence. It is interesting to speculate as to whether higher detection rates after this training might be associated with greater competence when fully trained as it does in other areas of endoscopy [35].

Despite the encouraging data that we have presented, there are some limitations when using ML in this setting. The software that is used to derive machine-based algorithms can sometimes be prone to “overfitting” or being specific to only the specific data analysed. Another problem of many of these methods is that they are often prone to finding spurious associations [36]. This means that although they perform well when applied to the original training set, they are not useful when applied to new data. Practically, this could be evaluated by expanding the current dataset to include new videos for external validation through asking users to score new lesions using our proposed decision tree. Lastly, all of the videos used in the study were taken from patients having an endoscopy performed using i-Scan image enhancement technology (PENTAX). This is still a relatively emerging technology compared to, for instance, NBI (Olympus) that is used in most endoscopy units. Therefore, unless the current training set is expanded to include videos of patients having endoscopy for BE with NBI (Olympus), the application of our algorithm to most hospitals would remain limited.

Although our algorithm appears to be more accurate than current classification systems, data for the two are not entirely comparable. Whereas existing classification systems only include patients with “flat” BE, high-definition endoscopy now allows the endoscopist to appreciate that many of these areas have subtle nodularity or ulceration [4, 37, 38]. We therefore also included patients with these lesions. We already know that the latter is more likely to harbour dysplasia or cancer and skew opinion. One could argue, however, that the inclusion of patients with “non-flat” BE is likely to be more representative of what is seen in day-to-day clinical practice.

Our classification system did not consider findings using ACA chromoendoscopy as no benefit was found when this was used. This is despite larger studies having demonstrated that the diagnostic accuracy is higher with targeted biopsies taken after ACA compared to a standard biopsy protocol [22]. More recently a two-staged training module was developed to evaluate the use of ACA for the detection of Barrett's neoplasia [39]. In this study, initial online training significantly improved the sensitivity of dysplasia detection from 83% to 95%. In contrast to our study, they then invited individuals to a one-day interactive seminar including live cases which further increased sensitivity of dysplasia detection to 98%. Furthermore, this group used 40 images and 20 videos to develop their training module compared to only 12 video cases of ACA in our study (although no difference in assessment between images and videos was observed). Based on these data, it would not be unreasonable to speculate that had a larger cohort of ACA data been used in our study, there is a greater likelihood that ACA would have been incorporated into a decision tree for dysplasia prediction.

## 5. Conclusion

ML is able to define rules learned from expert opinion. These generate a simple algorithm to accurately predict dysplasia. Once taught to non-experts, the algorithm significantly improves dysplasia detection rates. This opens the door to standardised training and assessment of competence for those who perform endoscopy in BE. It may shorten the learning curve and might also be used to compare competence of trainees with recognised experts for accreditation purposes.

## Figures and Tables

**Table 1:** Comparison of sensitivity and specificity between cases before and after acetic acid for all 3 experts. ND-BE: no dysplasia; D-BE: dysplasia.

	Expert 1 (%)	Expert 2 (%)	Expert 3 (%)	Total (%)
*Before acetic acid*				
Sensitivity, % (95% C.I.)	94 (71–99)	76 (50–93)	94 (71–99)	88 (76–96)
Specificity, % (95% C.I.)	87 (66–97)	87 (66–97)	83 (61–95)	86 (75–93)
Negative predictive value, %	99.6	98.5	99.6	86 (75–93)
*After acetic acid*				
Sensitivity, % (95% C.I.)	100 (48–100)	80 (28–99)	100 (48–100)	93 (68–99)
Specificity, % (95% C.I.)	71 (29–96)	85 (42–99)	57 (18–90)	71 (47–89)
Negative predictive value, %	100	98.8	100	71 (47–89)

**Table 2:** Accuracy, sensitivity, and specificity amongst both groups on non-experts before and after web-based training.

	Registrars (*n* = 13)	Medical students (*n* = 9)	Both groups (*n* = 22)
*Accuracy*			
Before training (%)	65	53	60
After training (%)	68	63	66
*p* value	0.07	<0.001	<0.001
*Sensitivity*			
Before training	71	83	76
After training	83	84	83
*p* value	<0.0001	0.44	<0.001
*Specificity*			
Before training	60	31	48
After training	57	49	54
*p* value	0.20	<0.0001	0.02

## Data Availability

All data discussed in this article is available in a Microsoft Excel spreadsheet from the corresponding author upon request.

## Abbreviations

OAC: Oesophageal adenocarcinoma
BE: Barrett's oesophagus
WLE: White light endoscopy
ML: Machine learning
HD: High definition
ACA: Acetic acid
ND-BE: Nondysplastic Barrett's oesophagus
D-BE: Dysplastic Barrett's oesophagus
SpRs: Specialist Registrars.
